# Elimination of cervical cancer as a public health problem—how shorter brachytherapy could make a difference during COVID-19

**DOI:** 10.3332/ecancer.2022.1352

**Published:** 2022-02-07

**Authors:** Aparna Gangopadhyay

**Affiliations:** Independent Practice, 377, M. B. Road, Panchanantala, Kolkata 700049, India

**Keywords:** brachytherapy, cervix uteri, COVID-19 pandemic, radiation dose fractionation

## Abstract

The World Health Organization has called for elimination of cervical cancer as a public health problem and has adopted strategies in this regard. However, the estimates for achieving the goals depend on the ability to provide timely treatment in a certain proportion of cases. The coronavirus disease 2019 pandemic has had a serious impact on healthcare delivery in many low and middle income countries (LMICs) with the highest burden of cervical cancer; funds and infrastructure are being reallocated to deal with the emergency, and cancer care has been seriously affected. In the absence of clear and reliable estimates, the exact extent of disruption remains unclear. It is, therefore, essential that pragmatic approaches are adopted to save lives. There has been considerable debate regarding the use of the 9 Gy × 2 fractions high dose rate brachytherapy schedule for the treatment of locally advanced cervical carcinoma. However, in LMICs with the highest global burden of locally advanced cervical cancer cases, radiation facilities have been using this fractionation schedule in many cases to deal with the overwhelming number of patients, who would have otherwise been denied timely treatment. In view of the current pandemic, and the difficulties in accessing and delivering timely healthcare, mortality owing to delayed treatment cannot be denied in LMICs, which already have underequipped healthcare facilities. Use of the shortest available fractionation schedule to provide timely treatment would serve to save more lives in regions with high incidence and mortality from the disease.

## The cervical cancer problem in low and middle income countries

Low and middle income countries (LMICs) are the major contributors to the global burden of cervical carcinoma, with most cases presenting at locally advanced stages; these countries also report the highest mortality rates from the disease [1]. The numerous challenges to the prevention, diagnosis and treatment of cervical carcinoma in these settings have considerable impact on the number of completed treatments and lives saved [2]. Along with the availability of other treatment modalities, radiotherapy capacity is limited owing to the lack of infrastructure, medical personnel and representative data [3]. In addition, repeated hospital visits are considerably inconvenient for most patients owing to difficulties in transportation and socioeconomic reasons [4, 5]. Moreover, high-quality health care is inequitably distributed in many LMICs, and socioeconomically disadvantaged and vulnerable patients often receive poor quality care, in terms of both competence of healthcare personnel and treatment experience [6].

## Issues with brachytherapy

Brachytherapy has been an integral component of radiotherapy for carcinoma of the cervix, as it improves radiocurability by allowing the delivery of high doses of radiation to the central tumour volume [7]. Although traditionally delivered by low dose rate equipment, the use of high dose rate (HDR) treatment delivery has become popular worldwide owing to the possibility of outpatient treatment, avoidance of radiation exposure to staff, consistent and reproducible applicator positioning and dose optimisation, achieved with a variable dwell-time stepping source [8]. In LMICs, there are substantial disparities between radiotherapy capacity and patient requirements. Brachytherapy services are difficult to access and are mostly located in larger urban centres. Patient waiting times are therefore long, making timely delivery of treatment an area of considerable concern. Owing to the greater possibility of normal tissue sparing with advancements in technology, it has been possible to use larger fraction sizes, which allows earlier treatment completion [9]. In low resource regions, where patient turnover rates and treatment compliance are major issues, hypofractionation is widely employed for increasing patient turnover and treatment compliance with encouraging results [10, 11].

Hypofractionated schedules serve to reduce the number of visits and increase patient turnover rates, both of which are particularly necessary in these settings. In view of the current coronavirus disease 2019 (COVID-19) pandemic, there have been difficulties in healthcare delivery worldwide [12, 13]. In particular, the already overburdened radiation therapy facilities in LMICs have been further affected by reallocation of funds and shortage of available staff [14, 15]. The use of hypofractionated schedules has, therefore, become particularly relevant in this scenario.

## Guidelines for dose-fractionation of HDR brachytherapy for cervical cancer

The American Brachytherapy Society (ABS) consensus guidelines for HDR brachytherapy in locally advanced cervical carcinoma recommend the administration of five or six fractions, delivered weekly [16]. A report by the ABS Task Group also suggests the use of smaller fraction sizes for reducing treatment toxicity [17]. However, a multi-institutional trial that compared the outcomes between HDR brachytherapy delivered in four 7-Gy and two 9-Gy fractions demonstrated similar overall survival and a comparable incidence of serious toxicities [18].

## Clinical use of the 9 Gy × 2 regimen

The LMICs in Southeast Asia remain the major contributors to the global burden of cervical carcinoma and also have the highest mortality rates from the disease [1]. In view of the limited infrastructure, shortage of trained staff and patient convenience, there has always been a compelling need to implement clinically feasible shorter fractionation schedules for HDR brachytherapy. As the shortest fractionation schedule, the 9 Gy × 2 regimen has, therefore, gained particular popularity in these regions, and numerous studies have demonstrated the safety and efficacy of this regimen [9–11]. In this context, it is noteworthy that many brachytherapy facilities in LMICs lack facilities for interstitial needle placement, solely depending on the use of intracavitary applications. In locally advanced cases, tumour regression following external beam radiotherapy is often delayed owing to the considerable burden of parametrial disease; clinicians in underequipped facilities, therefore, often wait for optimal tumour regression to ensure adequate coverage of the clinical target volume. In such cases, the 2-fraction regimen provides a pragmatic means of completing definitive treatment on schedule. However, the proposed recommendations published in view of the COVID-19 pandemic have advised against the use of the 9 Gy x 2 regimen, citing its radiobiological disadvantages [19].

## Impact of treatment on cervical cancer-related mortality in LMICs

The overall incidence of cancer is lower in LMICs compared to that of high-income countries; however, total cancer-related mortality is significantly higher in LMICs, especially among those younger than 65 years. The significant economic impact resulting from premature mortality and lost years of productivity is an area of considerable concern [20]. The United Nations sustainable development goals intend to achieve a one-third reduction in premature mortality from non-communicable diseases by 2030 [21]. As cervical cancer is both preventable and curable, the World Health Assembly passed a resolution in August 2020, calling for the elimination of cervical cancer as a public health problem, and also adopted a strategy to realise this goal [22]. This is first ever initiative for the elimination of a cancer worldwide.

The World Health Organization (WHO) has accordingly developed the WHO Cervical Cancer Elimination Modelling Consortium, involving three independent dynamic models of human papilloma virus infection, cervical carcinogenesis, screening and precancer and invasive cancer treatment. The triple-intervention strategy adopted by the WHO involves the use of vaccination strategies, screening and treatment for eliminating cervical cancer. It is expected that this strategy will prevent approximately a third of the premature deaths from cervical cancer in LMICs over the next 10 years, and nearly 90% of deaths over the next century.

A recent study estimated the reductions in age-standardised rates of cervical cancer mortality in 78 low-income and lower-middle-income countries for three core scenarios, namely, girls-only vaccination; girls-only vaccination with once-lifetime screening and cancer treatment scale-up; and girls-only vaccination with twice-lifetime screening and cancer treatment scale-up [23]. The study reported that the estimated mortality rate due to cervical cancer across all the 78 LMICs was 13.2 per 100,000 women in 2020. The findings suggested that compared to the status quo, vaccination alone would have minimal impact on cervical cancer mortality by 2030, with an estimated reduction of 0∙1%. However, additional scaling up of twice-lifetime screening and cancer treatment would reduce mortality by 34.2%, thereby preventing an estimated 300,000 deaths by 2030. It has been projected that twice-lifetime screening and cancer treatment would prevent 14∙6 million deaths by 2070, and 62∙6 million deaths by 2120.

The study concluded that the strategy may achieve a one-third reduction in the rate of premature mortality from cervical cancer in LMICs over the next 10 years, and will thus allow realisation of the 2030 United Nations sustainable development goals. In addition, the successful implementation of the elimination strategy proposed by the WHO would reduce mortality due to cervical cancer by nearly 99%, and save more than 62 million lives over the next century.

## Concerns in view of the COVID-19 pandemic

Notably, the mentioned study assumed that half of the patients with invasive cervical cancer would receive adequate treatment by 2023; this was expected to increase to 90% by 2030 [23]. However, there are no current estimates on the number of patients with cervical cancer who are being denied or are unable to access screening and treatment services during the COVID-19 pandemic. Nevertheless, recent reports from the Asia-Pacific region suggest that similar to other regions across the globe, cancer care is being considerably affected by the pandemic [24–26], with screening and treatment services being equally affected [27, 28]. A surge in cases has, therefore, been expected on resumption of usual services [29, 30]. In LMICs, where cancer care facilities are already overburdened, treatment delays are inevitable. Patient turnover, therefore, assumes particular importance in the successful reduction of mortality.

In the context of cervical cancer, most patients present in locally advanced stages in these settings [1]. The standard treatment, therefore, essentially comprises whole pelvic external beam radiation and concurrent chemotherapy [31], with brachytherapy as an integral component. In view of the paucity of brachytherapy facilities available in these settings, and the reallocation of funding during the pandemic, access to brachytherapy has been particularly restricted [32, 33].

Hypofractionation, therefore, represents a feasible approach for improving patient turnover. As the shortest available HDR fractionation schedule for locally advanced cervical cancer, the 9 Gy × 2 fractions regimen gains particular importance in saving lives.

A multicentre international clinical trial that recruited 601 patients, demonstrated no significant differences in terms of overall survival or severe treatment-related side effects between patients who received HDR brachytherapy with four fractions of 7 Gy and those who received two fractions of 9 Gy each [18].

In view of the prevailing difficulties in treatment during the pandemic, it is clear that the reduction of even a single extra fraction may offer definite benefits in terms of patient turnover in the overburdened brachytherapy facilities of LMICs [34].

## Case of a major global contributor: India

India is one of the major contributors to the global mortality from cervical cancer, accounting for nearly one-third of cervical cancer deaths worldwide. Women in this vast nation have a 1.6% cumulative risk of developing cervical cancer and a 1.0% cumulative risk of death from the disease [35]. A report on cancer statistics from the National Cancer Registry Programme in India indicated that most cervical cancer cases continue to present at locally advanced stages in the country, and that chemoradiation remains the commonest treatment administered [36]. In this context, it is worth noting that many cases are missed during data collection, as cancer is not a notifiable disease in India; actual incidence and mortality figures are therefore likely to be higher. Estimates from a study in India suggested that delays in the diagnosis and treatment due to the COVID-19 pandemic will increase the number of deaths from cervical cancer [37].

## Why a shorter brachytherapy schedule may make a difference

LMICs face serious constraints in radiotherapy capacity, delaying timely intervention in many cases. Brachytherapy is particularly resource intensive, and there are numerous financial and accessibility issues that hinder access to timely treatment in these settings. Use of the shorter 2-fraction regimen will reduce patient waiting times and increase the number of completed treatments within a given timeframe; this, in turn will translate to more lives being saved. In the face of overwhelming patient numbers, the shorter schedule will also improve treatment quality in the overburdened radiotherapy facilities of LMICs.

## Conclusions

Although overburdened brachytherapy facilities in LMICs have been using shorter fractionation schedules to ensure timely delivery of treatment to many patients with cervical cancer, the need for expediting treatment completion has never been as critical as in the era of COVID-19. The serious impact on screening and treatment accessibility implies that the number of locally advanced cases is set to rise in future; increased cervical cancer related mortality is therefore inevitable. Under the circumstances, shorter brachytherapy fractionation will serve as a notable life-saver in the difficult times of COVID-19 and offer a practical means for realizing the United Nations sustainable development goals.

## Funding

None.

## Conflicts of interest

None.
